# Bio- and toxic elements in edible wild mushrooms from two regions of potentially different environmental conditions in eastern Poland

**DOI:** 10.1007/s11356-016-7371-0

**Published:** 2016-08-11

**Authors:** Justyna Brzezicha-Cirocka, Małgorzata Mędyk, Jerzy Falandysz, Piotr Szefer

**Affiliations:** 1 Department of Food Science, Gdańsk Medical University, 107 Gen. J. Haller Av, 80-416 Gdańsk, Poland; 2 Laboratory of Environmental Chemistry and Ecotoxicology, Gdańsk University, 63 Wita Stwosza Str, 80-308 Gdańsk, Poland

**Keywords:** Foraging, Forest, Mushrooms, *Boletus*, *Cantharellus*, *Leccinum*, *Lycoperdon*

## Abstract

In the present study, the composition of bio-elements (K, Na, Mg, Ca, Fe, Cu, Zn) and toxic elements (Ag, Cd) in seven edible mushrooms from the rural and woodland region of Morąg (north-eastern Poland) and the rural and industrial region of the Tarnobrzeska Upland (south-eastern Poland) were investigated using a validated method. The species examined were *Boletus edulis*, *Cantharellus cibarius*, *Leccinum aurantiacum*, *Leccinum versipelle*, *Lycoperdon perlatum*, *Suillus luteus*, and *Xerocomus subtomentosus*. Final determination was carried out by flame atomic absorption spectroscopy (FAAS) after microwave-assisted decomposition of sample matrices with solutions of concentrated nitric acid in the pressurized polytetrafluoroethylene vessels. The contents of the alkali elements and alkali earth elements were determined in the species surveyed. The alkali elements, earth alkali elements, and transition metals (Ag, Cu, Zn, Fe, and Mn) were at typical concentrations as was determined for the same or similar species elsewhere in Poland and Europe. The results may suggest a lack of local and regional emissions of those metallic elements from industrialization of some sites in the Tarnobrzeska Plain. Cadmium was at elevated concentrations in *L. versipelle* from the Tarnobrzeska Plain but the reason—pollution or geogenic source—was unknown, while it was at typical concentrations in other species.

## Introduction

Wild-growing saprobic and symbiotic mushrooms may accumulate in their fruiting bodies’ considerable amounts of metallic elements and metalloids due to specificities in their physiology (Aloupi et al. 2011; Frankowska et al. 2010; Kalač 2016). Mushrooms are relatively rich in mineral constituents, both edible and inedible or poisonous species which all are eaten by game animals (wild boars, stags, and others) and may end up in humans via the food chain (Brzostowski et al. 2011; Falandysz et al. 2007a, b, 2015). This is spectacularly evidenced for radiocesium (^137^Cs), which is radiotoxic but also for typical toxic metals such as cadmium (Cd), lead (Pb), mercury (Hg), or silver (Ag), which are well bio-concentrated from soil by many mushrooms (Borovička et al. 2010; Falandysz and Brzostowski, 2007; Malinowska et al. 2006; Solomko et al. 1986; Vinichuk et al. 2010).

Edible wild-growing mushrooms with an estimated number of 2000 species worldwide are a portion of a larger group of similar species (fungi forming fruit bodies in the form of mushrooms or similar shapes which are also called sporocarps) in the Kingdom of Fungi, and more studies are needed to characterize their mineral and other compound contents and compositions (Kalač 2016). An initial step in getting insight into mineral content and composition of edible mushrooms from the wild and their potential to accumulate minerals is the examination of crude (raw) fruiting bodies and the underneath substrate—soil or other (Falandysz et al. 2003, 2012; Garcia et al. 2009; Mleczek et al. 2013; Lu and Liang, 2014; Sarikurkcu et al. 2012).

This study investigates the occurrence of metallic elements (Ag, Ca, Cd, Cu, Fe, Mg, Na, K, and Zn) in fruiting bodies of seven species of mushrooms which are edible and can be foraged in Polish forests. Mushrooms were collected from two spatially distant forested areas in the north-east and south-east of Poland. The forested areas in the north-east are considered pristine when compared with other regions of the country. For example, the moss *Pleurozium schreberi* collected in 1975–1995 and 2010 from the north-east and north of Poland was significantly less affected by airborne Cd, Cr, Cu, Fe, Ni, Pb, and Zn than from other regions, while most polluted was from the south (Grodzińska et al. 1999; Kapusta et al. 2014).

## Materials and methods

**Fig. 1:**
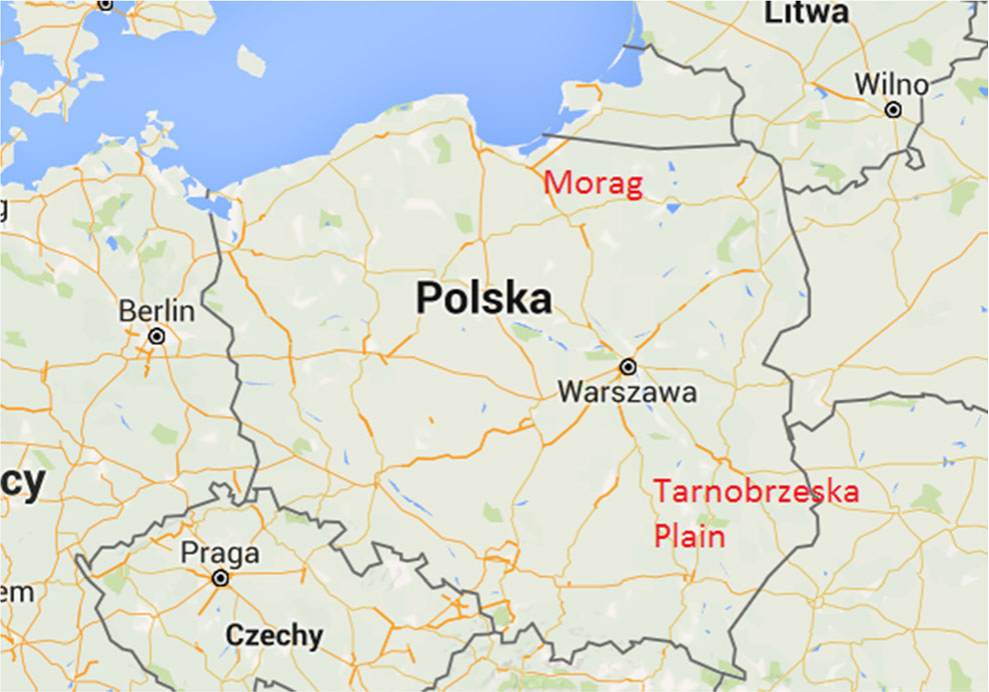
Location of the sampling sites: Morąg (coordinates 53° 54′ 58 57″ N 19° 55′ 40 25″ E) and Tarnobrzeska Plain (coordinates 50°35′ N 21°41′ E) in Poland (Google maps; color figure available online)

Fruit bodies of *Boletus edulis* Bull., *Cantharellus cibarius* Fr., *Leccinum aurantiacum* (Bull.) Gray, *Leccinum versipelle* (Fr. and Hök) Snell, *Lycoperdon perlatum* Pers., *Suillus luteus* (L.) Roussel, and *Xerocomus subtomentosus* L. were collected from the rural and woodland region near the town of Morąg in north-eastern Poland and from the Tarnobrzeska Plain (1411 km^2^) in south-eastern Poland, which is in part an industrial region with rural and forested areas (remainders of the Sandomierska Primeval forest) (Fig. 1.). In the Tarnobrzeska Plain region, the steel mill Huta Stalowa Wola is located in the town of Stalowa Wola (founded in 1938) and also the open-cast sulfur mine (also calcite, gypsum, quartz, aluminum oxide, celestine, and barite) in the Machów site (operated from 1964 to 1992) near the town of Tarnobrzeg.

Mushrooms (from 14 to 32 fruiting bodies per species) were cleaned from plant and soil debris, placed into plastic tray of an electrically heated commercial dryer, dried at 65 °C to constant mass and further ground in porcelain mortars. The subsamples of powdered fungal materials were digested with a solution of concentrated (65 %) nitric acid (Suprapur® Merck) in pressurized vessels made of polytetrafluoroethylene (PTFE) in an automatic microwave digestion system (MLS 1200). The metallic element concentrations were determined in an air-acetylene flame with the atomic absorption spectroscopy (AAS) method, using a background correction with a deuterium lamp. In order to determine the elements K and Na, cesium (Cs) salt was added to the samples as an ionization buffer at a concentration of 0.2 % *w*/*v*, and for Ca and Mg measurements, lanthanum (La) salt was added at a concentration of 0.1 % *w/v* as a releasing agent (Malinowska et al. 2004). Analytical control and analytical quality (AC/AQ) of determination were achieved by examination of procedural blanks and certified standard reference materials such as fish flour (SRM), lyophilized muscle tissue (MA-B-#/TM), and sea lettuce (BCR-279 IRMM *Ulva lactuca*). The results obtained after examination of the reference materials were satisfactory (Table 1). The computer software Statistica version 10.0 (Statsoft Polska, Kraków, Poland) was used for statistical analysis of data.

**Table 1:** Results (mg kg^−1^ dry biomass) of the measurements of accuracy of the analytical data using certificate reference materials CRM 279 *Ulva lactuca* (sea lettuce), SRM (fish flour), and MA-B-#/TM (lyophilized muscle tissue)

Element	Declared value	Own result	RSD (%)	Recovery (%)
Cd^a^	0.274 ± 0.022	0.232 ± 0.024	10.3	84.7
Cu^a^	13.14 ± 0.37	13.09 ± 0.23	1.65	99.6
Pb^a^	13.48 ± 0.36	13.10 ± 0.98	7.48	97.2
Zn^a^	51.30 ± 1.20	50.24 ± 1.59	3.16	97.9
Hg^b^	0.21 ± 0.02	0.26 ± 0.03	11.5	123.8
Fe^c^	95.4	92.5 ± 3.1	3.37	97.0
Mn^c^	2.62	2.39 ± 0.09	3.76	91.2
Ca^c^	3490	3456 ± 70	2.02	99.0
Mg^c^	1130	1040 ± 39	3.71	92.0
Na^c^	2160	1776 ± 53	2.97	82.2
K^c^	9320	9855 ± 452	4.59	105.7

^a^CRM 279 *Ulva lactuca* (sea lettuce)

^b^SRM (fish flour)

^c^MA-B-#/TM (lyophilized muscle tissue)

## Results and discussion

### Bio-elements

Baseline data on concentrations of the macro- and trace metallic elements determined in the mushrooms from the Morąg and Tarnobrzeska Plain sites are given in Table 1. The median values of the alkali element concentrations ranged from 27,000 to 47,000 mg kg^−1^ dry biomass (db) for potassium and from 57 to 260 mg kg^−1^ db for sodium. Hence, mushrooms foraged from the forests nearby to Morąg and from the Tarnobrzeska Plain, like many other mycorrhizal species collected in the wild from unpolluted areas, can be considered as a component of a diet that is rich in potassium and low in sodium.

Mushrooms as foodstuff can be a good source of minerals for human nutrition. However, minerals may leach out of the flesh of cooked mushrooms, e.g., into a liquid phase, when mushrooms are boiled at a short time (blanched), and the liquid is discarded. Without a doubt, all inorganic constituents well soluble in water will highly diminish in blanched mushrooms but the same will happen to some toxic elements, e.g., radiocesium. Another question is the bioavailability of minerals from mushrooms in the alimentary tract of man, which is a little-known process. It can be anticipated that mineral constituents easily leach out during blanching of fresh/frozen or cold soaking (macerating) of dried mushrooms, e.g., alkaline elements. In the absence of blanching or soaking, minerals in mushroom meals may be well bioavailable.

*B. edulis* from both sites showed in whole fruiting bodies similar concentrations of potassium—median values respectively at 27,000 and 31,000 mg kg^−1^ db (Table 2), which is consistent with data reported for caps and stipes of this species collected from other locations in Poland (Frankowska et al. 2010). In *L. perlatum*, potassium was at 28,000 mg kg^−1^ db which was similar to *B. edulis*. Both *C. cibarius* with potassium at 47,000 mg kg^−1^ db and *X. subtomentosus* with 46,000 mg kg^−1^ db in caps and 44,000 mg kg^−1^ db in stipes in this study can be considered as richer in this element than *B. edulis* and *L. perlatum*. Those data on potassium in *C. cibarius* and *X. subtomentosus* agreed with results from other studies (Falandysz and Drewnowska, 2015; Jarzyńska et al. 2012). The mushrooms of the genus *Leccinum* such as *L. aurantiacum* and *L. versipelle* were rich and similar in the content of potassium, which was, respectively, at 33,000 and 36,000 mg kg^−1^ db (median values). Those concentrations of potassium are consistent with the content in other *Leccinum* spp., e.g., *L. scabrum* (Bull.) gray (range of medians for caps at 34,000–52,000 mg kg^−1^ db) and *L*. *duriusculum* (Schulzer ex Kalchbr.) singer (median at 37,000 mg kg^−1^ db) (Falandysz et al. 2007c; Jarzyńska and Falandysz, 2012a). Potassium content in *S. luteus* with a median value in caps at 34,000 mg kg^−1^ db and in stipes at 35,000 mg kg^−1^ db was consistent with the median values for *S*. *grevillei* (range 27,000–40,000 mg kg^−1^ db for caps and 21,000–38,000 mg kg^−1^ db for stipes) (Chudzyński and Falandysz 2008). In view of the results obtained for potassium and in consideration of the literature data for the same species or genus, the industrialization of the Tarnobrzeska Plain was without an impact on the potassium content of mushrooms from the wild in this location.

**Table 2:** Trace elements in mushrooms from the region of Morąg (M) and Tarnobrzeska Plain (T) (mean value ± SD, median value—in parentheses—and range; mg kg^−1^ dry biomass)

Site	Species^a^	Element									
		K	Na	Mg	Ca	Cu	Zn	Fe	Mn	Ag	Cd
M	*Cantharellus cibarius* (W) *n* = 16 (162)	49,000 ± 4600 (47,000) 42,000–59,000	240 ± 58 (260) 140–360	1200 ± 130 (1100) 980–1400	1000 ± 260 (1000) 670–1500	54 ± 12 (52) 33–77	82 ± 9 (80) 69–100	330 ± 100 (330) 170–520	30 ± 10 (27) 20–63	0.53 ± 0.16 (0.55) 0.17–0.82	0.58 ± 0.05 (0.57) 0.49–0.67
M	*Lycoperdon perlatum* (W) *n* = 16 (138)	28,000 ± 2500 (28,000) 26,000–35,000	81 ± 20 (84) 47–120	1900 ± 250 (1900) 1700–2600	170 ± 160 (120) 68–730	110 ± 23 (100) 75–150	200 ± 23 (200) 170–240	500 ± 240 (460) 280–1200	46 ± 7 (46) 36–60	2.0 ± 0.6 (2.0) 1.1–3.9	2.2 ± 0.6 (2.4) 1.5–2.9
M	*Boletus edulis* (W) *n* = 32	27,000 ± 3000 (27,000) 21,000–31,000	360 ± 380 (210) 57–1400	910 ± 160 (890) 680–1300	480 ± 170 (460) 160–900	37 ± 16 (41) 15–70	160 ± 38 (160) 71–220	200 ± 150 (150) 51–610	21 ± 10 (20) 9.0–47	1.1 ± 0.8 (0.85) 0.16–3.1	2.8 ± 2.4 (2.2) 0.36–10
T	*Boletus edulis* (W) *n* = 29	32,000 ± 5600 (31,000) 24,000–41,000	190 ± 140 (160) 18–560	850 ± 100 (860) 680–1000	200 ± 53 (190) 110–300	36 ± 18 (35) 6.0–72	210 ± 43 (130) 130–320	47 ± 44 (35) 25–210	8.6 ± 3.7 (9.2) 4.0–15	WD	5.2 ± 4.7 (2.7) 0.33–18
M	*Leccinum aurantiacum* (W) *n* = 32	35,000 ± 7500 (33,000) 22,000–49,000	230 ± 170 (170) 19–680	1100 ± 380 (1100) 340–1900	580 ± 320 (520) 110–1600	41 ± 27 (37) 11–150	112 ± 76 (100) 20–320	270 ± 260 (150) 22–1100	24 ± 17 (17) 5.4–73	0.63 ± 0.63 (0.39) 0.18–3.3	0.81 ± 0.87 (0.56) 0.18–4.9
T	*Lecinum versipelle* (W) *n* = 29	41,000 ± 41,000 (36,000) 11,000–250,000	110 ± 52 (92) 10–250	1400 ± 1500 (1100) 440–9200	250 ± 94 (220) 110–490	36 ± 21 (30) 6.7–91	170 ± 110 (190) 16–550	92 ± 100 (47) 2.5–460	9.2 ± 3.7 (9.0) 4.2–19	WD	14 ± 14 (9.8) 0.97–57
T	*Suillus luteus* (C) *n* = 15	40,000 ± 13,000 (34,000) 23,000–75,000	100 ± 57 (84) 24–200	1300 ± 240 (1300) 970–1900	480 ± 160 (480) 320–980	20 ± 7 (17) 11–33	120 ± 30 (110) 78–180	360 ± 140 (360) 150–740	39 ± 11 (40) 24–62	WD	0.53 ± 0.19 (0.57) 0.19–0.80
T	*Suillus luteus* (S) *n* = 14	38,000 ± 18,000 (35,000) 12,000–71,000	110 ± 66 (98) 25–220	890 ± 360 (810) 430–2000	610 ± 250 (620) 220–1100	7.9 ± 4.0 (6.3) 4.0–17	49 ± 25 (43) 18–120	280 ± 20 (310) 120–540	40 ± 18 (35) 15–76	WD	0.58 ± 0.37 (0.48) 0.23–1.7
T	*Xerocomus subtomentosus* (C) *n* = 15	45,000 ± 10,000 (46,000) 11,000–53,000	54 ± 20 (57) 23–90	1200 ± 190 (1200) 610–1300	200 ± 140 (190) 47–500	24 ± 7 (25) 13–36	190 ± 47 (180) 94–300	190 ± 98 (200) 63–340	12 ± 6 (9.3) 6.7–27	WD	9.4 ± 3.6 (9.3) 3.6–16
T	*Xerocomus subtomentosus* (S) *n* = 15	43,000 ± 7500 (44,000) 30,000–59,000	59 ± 22 (58) 17–100	900 ± 200 (830) 540–1300	120 ± 62 (120) 20–230	16 ± 7 (16) 7.5–30	110 ± 40 (100) 58–180	68 ± 21 (63) 42–97	10 ± 5 (8.6) 4.3–19	WD	7.5 ± 3.2 (7.1) 3.3–14

*WD* without data

^a^Number of pooled samples and total number of fruit bodies (in parentheses); part of fruit body: *W* whole fruiting bodies, *C* caps, *S* stipes

Mushrooms are considered as a foodstuff very low in sodium (Vetter, 2003). In this study, the median values of sodium in a particular mushroom species or their morphological parts were below 300 mg kg^−1^ db, which is consistent with data reviewed recently for several species (Falandysz and Borovička, 2013).

Median values of magnesium and calcium, the alkali earth elements investigated in this study, were at a range of 810–1900 mg kg^−1^ db (Mg) and 120–1000 mg kg^−1^ db (Ca) (Table 2). They are both elements essential for biota, but mushrooms are usually substantially lower in calcium than in magnesium content (Falandysz and Borovička, 2013). The discrepancies in the median values of magnesium concentrations were negligible between the capped mushrooms, while the flesh of *L. perlatum* was more rich in this element and showing the maximum value mentioned earlier. Mushrooms in this study differed highly in concentrations of calcium concerning both different species but also individuals of the same species or species from the same genus as well as the morphological parts of fruit bodies (Table 2). Calcium as well as sodium, barium, and strontium occurred at greater concentrations in stipes than in caps of mature mushrooms (Falandysz and Borovička, 2013).

The transition metals such as Cu, Zn, Fe, and Mn are important cofactors in the enzymes of biota. A mushroom that is exceptionally rich in iron is *Suillus variegatus* (Sw.) Richon and Roze, with iron concentrations reported at a range of 3300–4100 mg kg^−1^ db (Falandysz et al. 2001). The range of the median values for iron in mushrooms surveyed was at 35–460 mg kg^−1^ db.

Mushrooms collected from the wild are usually rich in copper and zinc both of which are physiologically essential, while their concentrations differ for the species (Falandysz and Borovička, 2013). *L. perlatum* was richer both in Cu and Zn than other species examined, and the median values of their concentrations in caps or whole fruiting bodies were, respectively, at 100 and 16–52 mg kg^−1^ db for Cu, and at 200 and 80–190 mg kg^−1^ db for Zn. Stipes of *S. luteus* were much lower in Cu and Zn than caps (*p* < 0.05; Man-Whitney U test). Mushrooms of the genus *Boletus* are lower in copper than those of the genus *Agaricus* or *Macrolepiota* ((Alonso et al. 2003; Gucia et al. 2012a, b; Jorhem and Sundström, 1995; Mleczek et al. 2015). Copper in *C. cibarius* collected (median at 52 mg kg^−1^ db) and also zinc (median at 80 mg kg^−1^ db) were in typical concentrations for this species. For example, copper in *C. cibarius* from Spain was reported at 53–70 mg kg^−1^ db and from Sweden at 46 mg kg^−1^ db, while zinc, respectively, at 71–100 and at 110 mg kg^−1^ db (Alonso et al. 2003; Jorhem and Sundström, 1995).

*S. luteus* and *L. perlatum* showed manganese in fruiting bodies at 35–46 mg kg^−1^ db, and they were substantially richer in this element than other species in this study for which medians ranged from 8.6 to 27 mg kg^−1^ db (*p* < 0.05; Man-Whitney U test). Both the earth alkali elements and the transition metals (Cu, Zn, Fe, and Mn) in mushrooms were found at concentrations similar to those for the same or similar species elsewhere in Europe. The results may suggest a lack of local and regional emissions of those metallic elements from industrialization of some sites in the Tarnobrzeska Plain.

## Toxic elements

The toxic cadmium and silver determined in mushrooms in this study are both chalcophile elements, while the silver (Ag^+^) ion is highly proteotoxic. All specimens examined showed cadmium at a detectable concentration, while *X. subtomentosus*, *L. versipelle*, and *B. edulis* were substantially richer in this element than other species (Table 2). *C. cibarius*, *L. aurantiacum*, and *S. luteus* can be considered as low in cadmium (medians <1.0 mg kg^−1^ db). Exceptionally high in cadmium was *L. versipelle* from the Tarnobrzeska Plain for which the median value in whole fruiting bodies was at 9.8 mg kg^−1^ db. Currently, there is a lack of credible data on cadmium in *L. versipelle* from other regions of Poland, and it cannot be determined whether the measured cadmium values are geogenic or largely anthropogenic. Mercury in *L. versipelle* from the Tarnobrzeska Plain was at a similar concentration for this element to sites considered as uncontaminated from local or regional emission sources (Falandysz, 2002; Krasińska and Falandysz, 2016).

*Leccinum scabrum* sampled from the background (unpolluted) areas of Poland contained cadmium in caps at 2.4 to 5.7 mg kg^−1^ db (medians) (Falandysz et al. 2007c). Cadmium in *Leccinum griseum* (Quél.) Bresinsky & Manfr. Binder from a single site sampled in Poland was at 3.3 mg kg^−1^ db (median) in caps and at 1.2 mg kg^−1^ db (median) in stipes, and in *L. duriusculum*, respectively, at 1.3 db and 0.45 mg kg^−1^ db (Jarzyńska and Falandysz, 2012a, b). In light of those data on cadmium content of mushrooms of the genus *Leccinum*, the concentration recorded for *L. versipelle* from the Tarnobrzeska Plain seemed elevated, but more results from this region are needed to assess its possible anthropogenic origin or whether other factors are involved.

Cadmium and silver were at similar concentrations in *C. cibarius*, *L. perlatum*, and *L. aurantiacum* but silver was less abundant in *B. edulis* (Table 2). Silver and cadmium are efficiently accumulated from soil and other substrata by different mushrooms but fewer data are available on silver and a possible risk from this element in edible mushrooms and other foods than for cadmium (Byrne and Tušek-Žnidarič, 1990; Falandysz and Danisiewicz, 1995; Falandysz et al., 1994).

The maximum permissible concentration for cadmium in edible mushrooms set in the European Union is 0.2 mg kg^−1^ fresh product for three cultivated species such as *Agaricus bisporus* (J. E. Lange) Imbach, (common mushroom), *Pleurotus ostreatus* ((Jacq.) P. Kumm. (oyster mushroom), and *Lentinula edodes* (Berk.) Pegler (shiitake mushroom), and for other mushrooms, it is 1.0 mg kg^−1^ fresh product (Commission Regulation, 2008). There is a consensus that the moisture content of fresh mushrooms is at 90 %. The cited tolerance value for cadmium in mushrooms when expressed on a dry biomass basis is 2.0 mg kg^−1^ for cultivated species and 10 mg kg^−1^ for other mushrooms. In a light of those values, only *L. versipelle* from the Tarnobrzeska Plain showed cadmium in whole fruiting bodies at median concentrations close to the maximum value tolerated, while in other species, cadmium was much below this value.

## Conclusions

The alkali elements (K, Na), alkali earth elements (Ca, Mg), and transition metals (Ag, Cu, Zn, Fe, and Mn) in mushrooms were at concentrations similar to those determined for the same or similar species elsewhere in Poland and Europe. The results may suggest a lack of local and regional emissions of those metallic elements from industrialization of some sites in the Tarnobrzeska Plain. Cadmium was at elevated concentrations in *L. versipelle* from the Tarnobrzeska Plain but the reason—pollution or geogenic source—was unknown, while cadmium was at typical concentrations in other species.
